# Relationship of cell-free urine MicroRNA with lupus nephritis in children

**DOI:** 10.1186/s12969-016-0064-x

**Published:** 2016-01-14

**Authors:** Khalid M. Abulaban, Ndate Fall, Ravi Nunna, Jun Ying, Prasad Devarajan, Alexi Grom, Michael Bennett, Stacy P. Ardoin, Hermine I. Brunner

**Affiliations:** Cincinnati Children’s Hospital Medical Center, Cincinnati, OH USA; University of Cincinnati, College of Medicine, Cincinnati, OH USA; Ohio State University, Wexner College of Medicine, Columbus, OH USA; Division of Rheumatology, Helen Devos Children’s Hospital, 35 Michigan St NE, Suite 4150, MC081, Grand Rapids, MI 49503 USA

**Keywords:** SLE, Lupus nephritis, MicroRNA, Biomarker

## Abstract

**Background:**

MicroRNAs (miRNAs) are involved in the post-transcriptional regulation of genes. The objective of this study was to investigate whether select urinary cell-free microRNA’s may serve as biomarkers in children with active lupus nephritis (LN) and to assess their relationship to the recently identified combinatorial urine biomarkers, a.k.a. the LN-Panel (neutrophil gelatinase associated lipocalin, monocyte chemotactic protein 1, transferrin, and beta-trace protein).

**Methods:**

miRNAs (125a, 127, 146a, 150 and 155) were measured using real-time polymerase chain reaction in the urine pellet (PEL) and supernatant (SUP) in 14 patients with active LN, 10 patients with active extra-renal lupus, and 10 controls. The concentrations of the LN-Panel biomarkers (neutrophil gelatinase associated lipocalin, monocyte chemotactic protein-1, transferrin, beta-trace protein) was assayed. Traditional laboratory and clinical measures of LN and lupus (complements, protein to creatinine ratio; Systemic Lupus Erythematosus Disease Activity Index) were also measured.

**Results:**

All tested miRNAs in the SUP, but not the PEL, were associated with the LN-Panel biomarkers (0.3 < |r _Pearson_| < 0.73; *p* < 0.05), miRNA125a, miRNA127,miRNA146a also with C3 and dsDNA antibody levels (|r _Pearson_| > 0.24; *p* < 0.05), and miRNA146a with the renal domain of the SLEDAI (|r _Pearson_| = 0.32; *p* < 0.05). Mean miRNA levels of patients with active LN did not statistically (*P* > 0.05) differ from those of SLE patients without LN or controls.

**Conclusion:**

Levels of cell-free miR-125a, miR-150, and miR-155 in the urine supernatant are associated with the expression of LN-Panel biomarkers and some LN measures. These miRNA’s may complement, but are unlikely superior to the LN-Panel for estimating concurrent LN activity.

## Background

Lupus nephritis (LN) continues to result in a significant morbidity and mortality [1, 2]. Current laboratory and clinical measures of kidney involvement with systemic lupus erythematosus (SLE) have been found unsuited to monitor the course of LN accurately, making effective LN therapy virtually impossible [3]. Therefore, there is active ongoing research to identify improved measures of LN activity which led to the discovery of several combinatorial urinary protein biomarkers a.k.a. the LN-Panel, consisting of neutrophil gelatinase associated lipocalin (NGAL), monocyte chemotactic protein 1 (MCP-1), transferrin (Tf), and beta trace protein (BTP; a.k.a lipocalin-like prostaglandin synthase). Earlier studies suggest that the LN-Panel can be used to diagnose active LN, anticipate LN flares [4–6].

MicroRNAs (miRNAs), small non-coding RNAs about 20 nucleotides in length, are involved in the gene expression at the post-transcriptional level [7]. MiRNAs regulate an estimated 30 % of the human protein-coding genome, including the expression of genes involved in inflammation. Because miRNAs are characterized by high stability in tissues and body fluids and protected from endogenous RNAses, they are attractive candidate biomarkers. Prior studies showed that there is differential expression of miRNAs in the various renal compartments, enabling discrimination of glomerular from tubulointerstitial inflammatory changes [8]. In this setting prior reports suggested that especially miRNA’s 125a, 127, 146a, 150 and 155 may be differentially expressed with inflammatory kidney diseases, SLE or LN [9–11].

The objective of this pilot study was to explore the potential of select cell-free miRNAs (125a, 127, 146a, 150 and 155) when measured in urine to serve as LN biomarkers.

## Methods

With the approval of the institutional review board and after all the necessary consents and assents were obtained, we studied patients diagnosed with SLE prior to age 18 years [12] and a control with juvenile idiopathic arthritis (JIA) or fibromyalgia in this prospective cohort study. While SLE patients were studied every 6 months for up to three visits, controls were asked to participate in only one study visit.

At enrollment all SLE patient needed to have either active LN (LN-group) or active extra-renal SLE (SLE-group) as measured by the SLE Disease Activity Index (SLEDAI; score range of 0–105) [13]. Active LN was defined as a renal SLEDAI domain score of ≥ 8 in patients with biopsy-diagnosed LN (LN-Group), while active extra-renal SLE was defined presence of a SLEDAI score ≥ 8, excluding the renal domain items (proteinuria, hematuria, pyuria, cellular urine casts). Additionally, for including in the SLE-group, patients had to lack a history of LN and have minimum disease duration of two year.

### LN and SLE measures

At each study visit of SLE patients the SLEDAI was completed. The renal domain scores served as a clinical measure of LN activity (SLE-renal; score range 0–16) and the remaining items were used to calculate an extrarenal SLE activity score (SLEDAI-extrarenal; score range 0–89). We also measured the renal domain score of the BILAG index as previously reported (BILAG-renal, range 0–12) [14]. We also recorded complement C3 and C4 levels, anti-dsDNA antibodies titer, amount of proteinuria as estimated by the protein to creatinine (P/C) ratio in a random urine sample, serum creatinine, and the glomerular filtration rate (GFR) as estimated by the modified Schwartz formula [15].

### Laboratory assays for miRNA measurement and the LN-Panel

#### miRNA determination

A single dose of urine preservative (Norgen Biotek, Ontario, Canada) was added to each urine sample that was intended for miRNA measurement prior to centrifugation at 4000 g for 20 min to pellet the cells. We then separated the urine sample to urine supernatant (SUP) and urine pellet (PEL). Then the urine PEL and SUP samples were frozen within 2 h of urine collection and stored at −80 °C until the time of batch.

Five candidate miRNAs (125a, 127, 146a, 150 and 155 ) were selected based upon their proposed involvement in the pathogenesis of lupus and kidney disease [10]. The mirRNeasy Mini Kit (Qiagen, Valencia, CA) was used for miRNA isolation from both the SUP and the PEL. As a spike-in control, 5 μl of C. *elegans*-miR-39 were added to each 5 ml aliquot of SUP and PEL. This was followed by miRNA concentration measurements, using TaqMan miR Real time PCR technology (Life technologies, Carlsbad, CA). Till date, no reliable or validated endogenous control miRNA has been established to normalize for the miRNA content. We have used RNU48 as the housekeeping miRNA to severe as reference standard. The data were analyzed using the ∆∆CT method [16]. The miRNA levels and the analysis shown are unadjusted to the urine creatinine as adjusted analysis was the same.

#### LN-Panel biomarkers

We measured urinary concentrations of Tf and BTP by immunonephelometry (Dade Behring Prospect, Marburg, Germany). Intra and inter-assay coefficients of variation (CV) of this assay were 3.4 % and 2.5 % for Tf and 2.3 % and 6.5 % for BTP. Urine concentrations of MCP-1 were measured by enzyme-linked immunosorbent assay (ELISA) (Human MCP-1, R&D Systems, Minneapolis, MN, USA). The respective intra-assay and inter-assay CV was 5.0 % and 5.1 %. NGAL levels were quantified by ELISA (Kit 036; AntibodyShop, Grusbakken, Denmark). Intra and inter-assay CV of NGAL were 5.0 % and 5.1 %. Urine creatinine measurements were made using a modified Jaffe reaction, and microalbumin (MALB) was measured by immunoturbidimetry, both on a Dimension Xp and plus HM Clinical Analyzer (Siemens, Munich, Germany). Coefficients of variability for the creatinine measurements were 2.4 % (intra) and 4.2 % (total), and 2.9 % (intra) and 5.9 % (inter) for MALB. TGF-β [CV inter/intra: 2.6 %/8.3 %] was measured by ELISA (R&D Systems, Minneapolis, MN) after acid activation. Briefly, 20 μL of 1 N HCl was added to 100 μL of urine sample, mixed by inversion and incubated at room temperature for 10 min. Next, the acidified sample was neutralized by adding 20 μL of 1.2 N NaOH/0.5 M HEPES, then the assay was immediately run per manufacturer’s instructions [CV inter/intra: 2.0 %/7.8 %].

All the urine biomarkers were normalized to urine creatinine (in mg/mL). Laboratory personnel measuring the urinary biomarkers were blinded to the clinical information.

### Statistical analysis

Urine levels of the LN-Panel biomarkers and the miRNAs were considered primary measures in this study. They were log-transformed in order to fit major assumptions of parametric statistical models in analyses. For each miRNA measure, its change over time was assessed using a mixed-effect model, adjusting for controlling covariates, mainly the demographics. Other numerical variables were summarized by geometric mean ± SE (standard error) values, and binary or categorical variables were summarized with frequency values (in %). Groups of patients were assessed for statistically significant differences using analysis of variance (ANOVA). Associations between miRNA measures, LN-Panel markers and other LN measures (SLEDAI, complement levels, GFR, serum creatinine) were assessed using Pearson’s correlation coefficients (r) which can be interpreted as follows: between 0.2 and 0.39 is considered to be weak, 0.4 and 0.59 is considered moderate, 0.6 and 0.79 is strong, and 0.8 to 1.00 is a very strong correlation [17]. Excel XP (Microsoft, Redmond, WA) and SAS 9.4 (SAS Institute, Cary, NC) programs were used for analysis. Two-sided *P* values less than 0.05 were considered significant.

## Results

### Characteristics of patients

The demographic information of the participants is summarized in Table 1. There were 39 visits (baseline and follow up visits) for the LN-group and 29 visits for the SLE-group available for analysis. Majority of the LN patients had Class 4 (50 %) followed by Class 2 (28.6 %) as per the International Society of Nephrology/Renal Pathology Society Classification for LN [18]. Controls with JIA were treated with non-steroidal anti-inflammatory medications (*n* = 2), methotrexate (MTX, *n* = 2) or etanercept plus MTX (*n* = 1). None of the fibromyalgia patients was treated with anti-inflammatory medications.

**Table 1:** Demographics and clinical information

Features		*n* of *N* (%)	Mean (SD)
Lupus patients Controls	Lupus Nephritis (LN),n, age (years)	14 18.33 ± 3.77	
	SLE without LN,n, age (years)	10 17.40 ± 2.59	
	Fibromyalgia	5	
	Juvenile Idiopathic Arthritis	5	
Females		19 (79.1 %)	
Race	Black	13 (54.1 %)	
	White	8 (33.4 %)	
	Other°	3 (12.5 %)	
Medications (SLE patients)	Oral prednisone (mg/day)	24 (100 %)	26.1 (18.4)
	Mycophenolate mofetil	10 (41.7 %)	
	Azathioprine	1 (4.1 %)	
	Cyclophosphamide	10 (41.7 %)	
	Diuretics	4 (16.6 %)	
	Angiotensin system blocking drug	12 (50 %)	
LN Status (*n* = 14)	GFR^£^ < 60 ml/min/m^2^	3 (21.4 %)	
	Protein:creatinine ratio > 0.5	11 (78.6 %)	
	Renal SLEDAI^*^ score		5.6 (6.19)
	Renal BILAG score		4.8 (4.54)
	Class 2	4 (28.6 %)	
	ISN/RPS^¶^ Class 3	2 (14.3 %)	
	Class 4	7 (50 %)	
	Class 5	1 (7.1 %)	
SLE Status (*n* = 24)	Complement C3 low	9 (37.5 %)	
	Complement C4 low	16 (66.7 %)	
	Presence of anti-dsDNA antibodies	18 (75 %)	

*Systemic Lupus Erythematosus Disease Activity Index, range 0–105; 0 – inactive LN

¶International Society for Nephrology Renal Pathology Society Class; there was no biopsy consistent with Class I or VI; °Other: includes Asians, Mixed race, Native Indian

^£^GFR as estimated by the modified Schwartz formula

### Levels of candidate miRNAs

The mean urine concentrations of the miRNAs considered were statistically significantly higher in the PEL than the SUP in all groups, including controls (*p* < 0.05). However, as is summarized in Table 2, there were no significant (*P* > 0.05) differences in any of the miRNA concentrations between the SLE-group and the LN-group, or controls, irrespective of whether considering the supernatant or the pellet. There was merely a trend towards higher levels of miR-127 in the SUP of the SLE-group compared to the LN-group. Notably, miRNA levels lacked association with patient age, sex and race (*p* > 0.05).

**Table 2:** Mean levels of the urinary miRNA’s in the supernatant and pellet in different groups at visit 1

	Mean ± SE^a^			*P* value^b^
	Controls^c^ (*n* = 10)	SLE Group (*n* = 10)	LN Group (*n* = 14)	
Urine Supernatant				
miR125a	5.83 ± 0.59	5.67 ± 0.53	6.66 ± 0.48	NS
miR127	6.65 ± 0.60	5.24 ± 0.54	5.26 ± 0.51	NS
miR146a	7.98 ± 0.93	7.61 ± 0.84	7.15 ± 0.76	NS
miR150	4.08 ± 0.73	4.08 ± 0.65	4.88 ± 0.62	NS
miR155	4.72 ± 0.64	4.03 ± 0.58	5.23 ± 0.53	NS
Urine Pellet				
miR125a	9.45 ± 0.67	9.89 ± 0.60	10.21 ± 0.55	NS
miR127	8.87 ± 0.69	9.10 ± 0.62	8.59 ± 0.56	NS
miR146a	11.02 ± 0.52	10.74 ± 0.44	10.77 ± 0.40	NS
miR150	9.14 ± 0.62	9.34 ± 0.55	9.89 ± 0.53	NS
miR155	9.20 ± 0.62	9.10 ± 0.56	10.01 ± 0.51	NS

Values of miRNA’s are relative expression normalized to RNU48

*miR* micro RNA, *JIA* Juvenile Idiopathic Arthritis, *LN* Lupus Nephritis

^a^SE, standard Error

^b^ *P*-values based on a *t*- test comparing means of all different groups , NS, not significant with p-value >0.05

^c^Controls include fibromyalgia and the Juvenile idiopathic arthritis patients together and their values were almost identical

### Association of candidate miRNAs with other LN measures

When measured in the PEL all of the miRNA had weaker association to all of the LN and SLE measures than miRNAs measured in the SUP. Table 3 summarizes associations between miRNA levels in the SUP with SLE and LN outcomes (Table 3). All of the LN-Panel biomarkers were positively associated with SUP values of miR125a and miR150 (0.4 < r ≤ 0.73; *P* < 0.05), while miRNA127 and miRNA155 lacked association with NGAL but still were weakly to moderately associated with the other LN-Panel biomarkers (TF, BTP, MCP-1).

**Table 3:** Associations of MiRNA and LN-Panel levels in the urine supernatant vs. traditional measures^a^

Variables^b^	miR125a	miR127	miR146a	miR150	miR155	Tf	BTP	NGAL	MCP-1
LN-Panel									
Transferrin (Tf)	0.44	−0.24	-	0.65	0.54	1.0	-	0.42	0.27
BTP	0.73	−0.24	−0.37	0.68	0.35	-	1.0	0.43	0.21
NGAL	0.48	-	−0.27	0.39	-	-	-	1.0	0.58
MCP-1	0.44	0.34	-	0.45	0.68	-	-	-	1.0
WBC (k/mcL)	0.23	-	-	0.40	-	0.30	0.25	-	0.37
Hemoglobin (mg/dL)	-	-	-	-	-	−0.21	0.42	-	-
Platelets count (k/mcL)	−0.39	−0.24	-	-	-	-	0.25	−0.27	−0.21
ESR (mm/h)	-	0.24	-	-	-	-	0.25	-	0.36
Anti-dsDNA titer	-	−0.31	−0.28	-	−0.27		-	0.5	0.36
C3 level (mg/dL)	−0.32	-	0.24	--	−0.36	0.23	−0.43	−0.41	−0.2
C4 level (mg/dL)	-	-	-	-	−0.29	-	−0.26	−0.31	−0.21
Urine P/C ratio	-	-	-	0.38	0.36	-	-	-	-
GFR	-	-	-			−0.21			
Renal - SLEDAI^d^	-	-	−0.32	-	-	0.46	0.30	0.53	0.49
Renal BILAG^e^	0.29	-	0.29	0.27	0.20	0.46	0.26	0.48	0.31
Extrarenal SLEDAI^c^	-	-	-	-	-	-	-	-	-

^a^Only statistically significant associations with r values ≥ 0.20 are shown; “-“means, no statistically significant correlation was found (*p* > 0.05)

^b^Values are Pearson correlation coefficients of log-transformed urine concentrations the miRNAs listed with *miR* micro RNA, *BTP* beta trace protein, *NGAL* neutrophil gelatinase associated lipocalin, *MCP monocyte chemotactic protein 1*, *WBC* white blood count, *DSDNA* anti-double stranded DNA titer, *GFR* glomerular filtration rate, *P/C* protein to creatinine

^c^SLEDAI, Systemic Lupus Erythematosus Disease Activity Index, range 0–105; 0 inactive LN

^d^Renal-SLEDAI, renal domain of the Systemic Lupus Erythematosus Disease Activity Index

^e^Renal-BILAG, renal domain of the British Isles Lupus Activity Group Index

Besides miRNA127, all of the other miRNA when measured in the SUP were associated with LN activity (SLEDAI-renal; BILAG-renal), but correlations where generally weaker than those of the LN-Panel biomarkers (Table 3). Notable neither SUP miRNA levels nor the LN-Panel biomarkers were related to SLEDAI-extrarenal scores.

### MiRNA levels and the course of LN

When examining miRNA levels over the course of LN activity (renal-SLEDAI), only miR-146a in the SUP showed a trend towards lower levels with worsening of LN activity, and a trend towards higher levels with improving LN activity (both *P* > 0.05). None of the other miRNAs (miR125a, miR 127, miR 150, miR 155) was found to have a consistent trend with the course of activity.

## Discussion

We found closer relationships between the levels of cell-free miRNAs when measured in the urine supernatant than the pellet with the presence of LN. Notably, the urine concentrations of previously described protein biomarkers a.k.a. the LN-Panel were more closely related to the clinical presentation of LN than the cell-free miRNA biomarkers considered in this pilot study.

Though the miRNAs 125a, 127, 146a, 150 and 155 are produced by various kidney cells and free miRNA may stem from circulation or urine, we failed to document strong association with these miRNAs in the pellet with either the extra-renal SLEDAI, renal –SLEDAI or the traditional laboratory measures.

A potential explanation for observing some relationships of miRNA’s with LN activity in the supernatant, but not the pellet is that these detected miRNA in the pellet may not reflect miRNA from the kidney, but rather from cells of the external urinary canal. For that reason, we advocate that measurement of miRNA’s in the urine should be done from supernatant and not the pellet. We found urine miR-127 to be higher in the LN group compared to the SLE group. Although this difference was not statistically significant, our findings were similar to Dai R et al. who reported miR-127 to be upregulated in the splenocytes of Lpr mice [19]. More studies are still needed to fully understand the significance of miR-127 in LN.

Bench studies showed that the select miRNA have crucial roles in regulating the immune response in the pathogenesis of LN, either negatively like miR-146a and 155 or positively like miR-150 [10, 20]. When examining these miRNAs for their potential to serve as clinical biomarkers in the urine, our cross-sectional and longitudinal data indicate that only miR-146a had a weak association with the renal–SLEDAI and changed with the course of LN activity. This is in line with some previous studies [20, 21]. However, none of the other miRNA measured were associated with clinical or laboratory measures of LN activity.

We have shown that the levels of miR125a, miR150 and miR155 had associations with the levels of the LN-panel biomarkers namely BTP, MCP-1 and Tf. These protein biomarkers have been proven to be accurate biomarkers of LN activity in previous studies [6, 22]. Possible hypotheses to the association that we see between select miRNA’s and the LN-Panel that these three miRNA’s may have an important role in regulating the LN –Panel protein biomarkers or indeed associated with LN activity, but due low subject number , no significant association was found. Our results indicate that these selected cell-free miRNAs are unlikely superior to the LN-Panel as clinical biomarkers for LN activity, and may be inferior based on performance of the LN-Panel of having stronger associations with the LN activity compared to the selected miRNA’s.

Also extracting the miRNAs from the urine compared to the LN-Panel markers, are time consuming and assays have not yet been standardized.

Some limitations of this study are there could be some racial or gender differences that has been previously reported, but we were not able to find them possibly due to low subjects included [23]. Also our study examined the role of only five miRNA’s and the values of other miRNAs at large to serve as biomarkers for LN cannot be fully excluded. Nonetheless, our study is still valuable as we tested five highly relevant miRNAs reported for LN. For future studies on miRNA as biomarkers, the use of the high-throughput capacity technology offered by arrays will be an excellent approach option, since hundreds of miRNAs can be studied simultaneously. Another limitation is till date, there has been no reliable or validated endogenous control miRNA established to normalize for the miRNA content in the urine. We used RNU48, as it has been found to be a suitable housekeeping miRNAs in urothelial and endometrial carcinomas and most likely it will be suitable in the urine as well [24, 25] .

Our miRNA levels in the SUP and the PEL were generally very low compared to those usually found in the blood. To be able to isolate larger amount of miRNA’s in the urine we conducted a small pilot study (*n* = 7), using similar patients, but instead of extracting free floating total RNA from urine, we extracted them from exosomes by differntial centrifugation of the urine . Exosomes are small membrane vesicles with a size of 30–120 nm that are released by different cell types. Exosomes can be isolated from various body fluids. Lin-Li Lv et al. found that the detection of miRNA from the exosomes were stable and reproducible [26]. In line with this report, our pilot data support that harvesting miRNA from urine exosomes yields considerable higher amounts of miRNA than the cell-free urine, and by doing so we may be able to detect better and more valid associations.

## Conclusions

Translational research on miRNA in LN is still in its infancy. Our study in testing these five urinary cell-free miRNA’s (125a, 127, 146a, 150 and 155) to serve as biomarkers in LN appears less promising despite the proven relationship of these miRNA’s to the pathogenesis of SLE. Still further studies are needed and necessary to include a more differentially regulated miRNA in patients with LN and to examine their correlation in the urine.

## Abbreviations

BTP: beta-trace protein
CV: coefficients of variation
JIA: juvenile idiopathic arthritis
LN: Panel Lupus Nephritis Panel
LN: Lupus Nephritis
MCP1: monocyte chemotactic protein 1
miRNA: MicroRNA
NGAL: neutrophil gelatinase associated lipocalin
PEL: pellet
SLE: Systemic Lupus Erythematosus
SLEDAI: SLE Disease Activity Index
SUP: supernatant
Tf: transferrin
